# Adenomyosis with uterine abscess formation treated by adenomyomectomy: A report of two cases

**DOI:** 10.1111/jog.16185

**Published:** 2024-12-12

**Authors:** Daiki Hiratsuka, Chihiro Ishizawa, Rei Iida, Yamato Fukui, Mitsunori Matsuo, Masato Nishida, Masako Ikemura, Miyuki Harada, Osamu Wada‐Hiraike, Yutaka Osuga, Yasushi Hirota

**Affiliations:** ^1^ Department of Obstetrics and Gynecology, Graduate School of Medicine The University of Tokyo Tokyo Japan; ^2^ Department of Obstetrics and Gynecology National Hospital Organization, Kasumigaura Medical Center Kasumigaura Japan; ^3^ Department of Pathology, Graduate School of Medicine The University of Tokyo Tokyo Japan

**Keywords:** abscess, adenomyosis, dysmenorrhea, fertility, uterine bleeding

## Abstract

Uterine adenomyosis is a common disease in women of reproductive age that causes dysmenorrhea, abnormal uterine bleeding, infertility, and obstetric complications. Rarely, adenomyosis can lead to abscess formation, which is refractory to antibiotics and occasionally requires surgical treatment, such as hysterectomy. However, hysterectomy should be avoided in patients who seek to preserve fertility. Herein, for the first time, we report two cases of uterine adenomyosis with abscess formation during assisted reproductive procedures that were successfully treated with adenomyomectomy, thereby preserving fertility. A history of repeated intrauterine procedures and infections was crucial in making an appropriate preoperative diagnosis. Adenomyomectomy can be an effective treatment for adenomyosis associated with abscess in patients who wish to preserve fertility.

## INTRODUCTION

Adenomyosis is a common benign, hormone‐dependent disease that occurs in women of reproductive age. It is characterized by the growth of tissues resembling the endometrium within the myometrium of the uterus^1^ and causes symptoms such as dysmenorrhea and abnormal uterine bleeding, and infertility and obstetric complications.^1^, ^2^, ^3^ Cases of adenomyosis complicated by infections with pyometra have been reported. In such cases, uterine abscesses are often refractory to treatment, do not improve with antibiotics, and require hysterectomy.^4^, ^5^, ^6^, ^7^ However, hysterectomy should be avoided in patients with adenomyosis and uterine abscess who wish to preserve fertility. In this report, for the first time, we describe two cases of adenomyosis complicated by uterine abscess formation that were successfully treated with adenomyomectomy as a fertility‐preserving surgery in patients who underwent assisted reproductive procedures. The procedure of obtaining written informed consent was substituted with an informed opt‐out procedure. The publication of this case report was approved by the Research Ethics Committee of the Faculty of Medicine at the University of Tokyo. The procedures described in this case report adhered to the principles of the Declaration of Helsinki.

## CASE PRESENTATION

### Case 1

**FIGURE 1:**
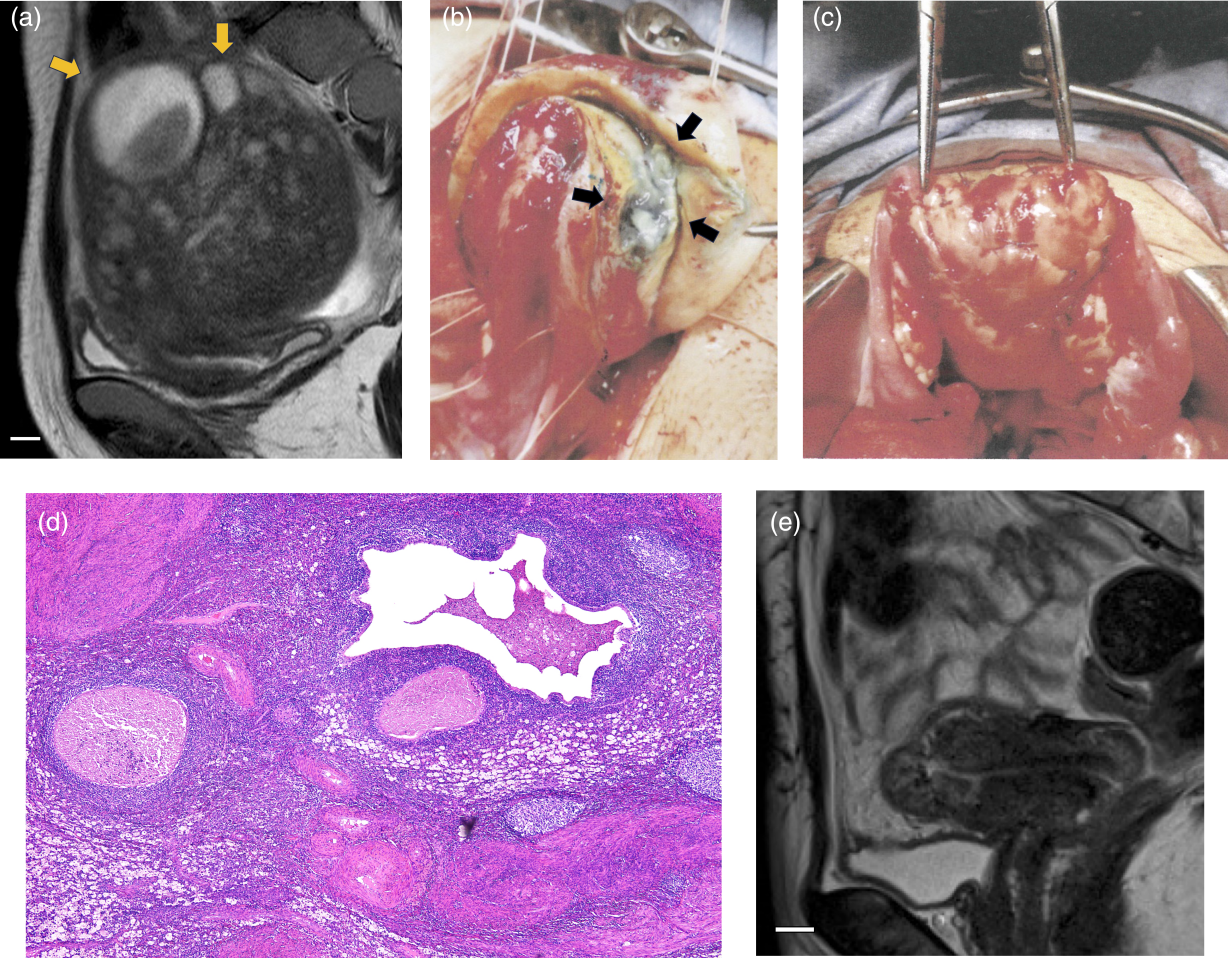
Findings in case 1. (a) Preoperative T2‐weighted sagittal magnetic resonance image. The arrow indicates cysts within the myometrium showing high signal intensity. The uterus has diffused adenomyosis. The scale bar indicates 1 cm. (b) Laparotomy findings. The arrow indicates a cystic lesion within the myometrium. When the cyst was ruptured, the contents of the abscess spilled out. (c) Postoperative laparotomy findings. (d) Hematoxylin and eosin staining of the specimen. Infiltration of neutrophils and macrophages, and granulomatous inflammation were observed (×4). (e) Postoperative T2‐weighted sagittal magnetic resonance image. The scale bar indicates 1 cm.

A 31‐year‐old woman (gravida 1, para 0) with infertility and dysmenorrhea was admitted to our hospital. She had a history of undergoing transcervical resectoscopy for endometrial polyps. On admission, we observed diffuse uterine adenomyosis involving both the anterior and posterior uterine walls using transvaginal ultrasonography. Contrast‐enhanced magnetic resonance imaging (MRI) revealed a uterus measuring 62 × 62 × 64 mm. No abnormal findings were observed on other screening examinations for infertility. We administered eight courses of a gonadotropin‐releasing hormone agonist (1.88 mg) subcutaneously and performed four embryo transfer procedures over 2 years; however, the patient could not achieve pregnancy. At 33 years of age, while undergoing assisted reproductive procedures, her dysmenorrhea became severe and was associated with fever. MRI re‐examination suggested the presence of a 55 × 48 × 38‐mm cyst and 24 × 16 × 12‐mm cyst in the anterior wall (Figure 1a). Endometrial biopsy revealed that the endometrium was in the proliferative phase with lymphoplasmacytic inflammation, suggesting infection. Laboratory findings included a white blood cell (WBC) count of 15 300/μL and a serum C‐reactive protein (CRP) level of 3.86 mg/dL. The patient was diagnosed with adenomyosis with abscess formation. Adenomyosis appeared to be the scaffold for infection, and not only drainage but also removal of the adenomyosis lesion were considered necessary to improve the symptoms. Laboratory findings after administration of levofloxacin 500 mg per day orally for 2 weeks included a WBC count of 5200/μL and a serum CRP level of 0.91 mg/dL. Adenomyomectomy was considered as a treatment for dysmenorrhea and adenomyosis. The patient was referred to another hospital, where an adenomyomectomy was performed. The uterine myometrium of the anterior and posterior walls and the fundus was incised to confirm a full‐layer lesion of adenomyosis. The greenish‐white abscesses within the cystic lesions were drained when the lesions were incised (Figure 1b). The abscess and surrounding lesions of adenomyosis were removed as much as possible with the loop electrosurgical excision procedure, while ensuring that the myometrium remained intact. The anterior and posterior walls were closed with double‐layer interrupted sutures, and the fundus was closed with triple‐layer interrupted sutures, using 0‐vicryl (Figure 1c). The total operative time was 2 h 50 min, and the volume of blood lost was 304 mL. The resected adenomyosis tissue weighed 88 g. The postoperative course was uneventful. Histopathological examination revealed uterine adenomyosis with degeneration, abscess formation, and granulomatous inflammation (Figure 1d). Postoperatively, the patient had no dysmenorrhea. Ten months postoperatively, we assessed a pelvic MRI and determined that it was safe for the patient to restart fertility treatments (Figure 1e). Twelve months postoperatively, the patient became pregnant spontaneously before she was provided with permission to conceive. At 13 weeks of pregnancy, the uterine myometrium at the fundus, where the abscess within the adenomyosis was formerly located, was only 4‐ to 7‐mm thick. The patient was diagnosed with total placenta previa and suspected placenta accreta at 26 weeks of pregnancy. The patient developed uterine rupture at the fundus at 30 weeks and 6 days of pregnancy, and an emergency cesarean section with supracervical hysterectomy was performed. The placenta accreta spectrum was observed pathologically. The baby was born weighing 1629 g and discharged without complications at 43 days of age.

### Case 2

**FIGURE 2:**
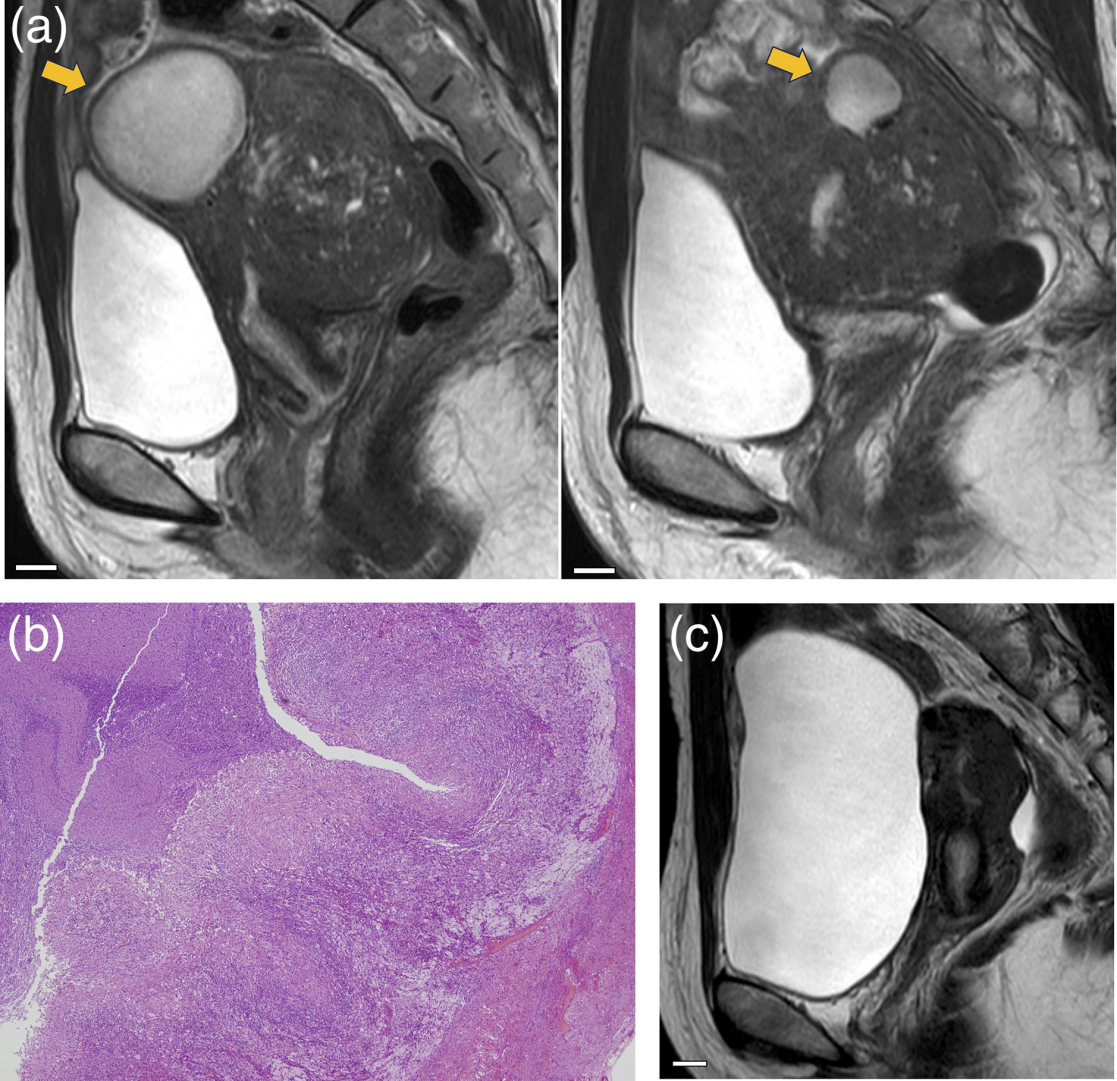
Findings in case 2. (a) Preoperative T2‐weighted sagittal magnetic resonance image. The arrow indicates cysts within the myometrium showing high and low signal intensities. The uterus has diffused adenomyosis. The scale bars indicate 1 cm. (b) Hematoxylin and eosin staining of the specimen. Infiltration of neutrophils and macrophages, and granulomatous inflammation were observed (×4). (c) Postoperative T2‐weighted sagittal magnetic resonance image. The scale bar indicates 1 cm.

A 41‐year‐old woman (gravida 0, para 0) with infertility and dysmenorrhea was admitted to our hospital. She had a history of undergoing transcervical resectoscopy for endometrial polyps. She underwent seven embryo transfer procedures; however, she could not achieve pregnancy. In addition, she experienced recurrent lower abdominal pain caused by uterine infection, which often required the administration of tazobactam/piperacillin intravenously. On admission, MRI revealed a 42 × 42 × 35‐mm cyst and 20 × 19 × 17‐mm cyst in the fundus and posterior wall of the uterus, respectively, and diffuse uterine adenomyosis of the posterior wall (Figure 2a). As in case 1, it was considered necessary to perform not only drainage but also surgical removal of the adenomyosis to improve symptoms. The patient strongly desired an adenomyomectomy to treat dysmenorrhea and infertility. Adenomyomectomy was performed after obtaining consent from the patient after informing her regarding the risks associated with the procedure, including uterine rupture during pregnancy. The uterine myometrium of the anterior and posterior walls and fundus were incised to confirm a full‐layer lesion of adenomyosis. Cystic lesions were observed in both the fundus and posterior wall of the uterus with adenomyosis, consistent with the MRI findings. When resecting these lesions, the cysts ruptured, and the greenish‐white contents of the abscess spilled out. The abscess and surrounding lesions of adenomyosis were removed as much as possible with the loop electrosurgical excision procedure, while ensuring that the myometrium remained intact. The myometrium was closed with triple‐layer interrupted sutures, using 0‐vicryl. The total operative time was 3 h and 38 min, and the volume of blood lost was 380 mL. The resected adenomyotic tissue weighed 25 g. The postoperative course was uneventful. Histopathological examination revealed uterine adenomyosis with cystic changes. The results of the culture test of the abscess showed that nothing was cultured; however, the cysts were abscesses with no epithelial lining and lined with neutrophils and inflammatory cells, indicating granulomatous inflammation (Figure 2b). Postoperatively, the patient had no dysmenorrhea. We performed MRI and a hysteroscopy to check the postoperative uterus and provided permission to conceive 6 months after the operation (Figure 2c).

## DISCUSSION

We encountered two cases of uterine adenomyosis with abscess formation that were treated with adenomyomectomy. While several cases of adenomyosis with uterine abscess formation have been reported, this is the first report of cases in which the uterus was preserved while treating uterine abscesses with adenomyomectomy.

Susceptibility to infection has been reported in cases of endometriosis.^8^, ^9^ In contrast, the risk of intrauterine infection in patients with adenomyosis remains unclear. This risk may be high in patients undergoing infertility treatment involving frequent intrauterine manipulations, such as in vitro fertilization, endometrial biopsy, and hysteroscopic surgery. In rare cases, an abscess develops for which antibiotics are less effective, and the infection may progress to disseminated intravascular coagulation or sepsis.^4^, ^5^, ^6^, ^7^ In previously reported cases of adenomyosis with abscess formation that was refractory to antibiotics,^4^, ^5^, ^6^, ^7^ hysterectomy would generally be necessary and was selected over simple drainage if there was no desire to preserve fertility. However, in both our cases, the patients wished to preserve their fertility and the infection was localized to the uterus, so we chose adenomyectomy as a uterine‐sparing procedure. Nonetheless, even if fertility preservation is desired, hysterectomy may be necessary if infection‐related factors such as disseminated intravascular coagulation cannot be controlled^7^ or if abscess formation involves the entire uterus. Therefore, appropriate diagnosis and treatment are necessary, especially when the abscess or infection is complicated and fertility preservation is required. Therefore, in cases similar to those presented in this report, adenomyomectomy may be a useful treatment for uterine adenomyosis with abscess formation in patients who wish to preserve fertility.

Appropriate diagnosis of abscess formation in uterine adenomyosis depends on imaging and interpretation of the clinical course. The diagnosis of uterine adenomyosis is typically based on MRI or ultrasonography, which are considered the standard imaging modalities.^10^ MRI is usually more specific and accurate than ultrasonography.^3^ MRI typically detects adenomyosis as a myometrial mass with indistinct margins and primarily low signal intensity or diffuse or focal thickening of the junctional zone, forming an ill‐defined area of low signal intensity on T2‐weighted images.^10^ A uterine abscess typically appears as a localized fluid collection within or around the uterine tissue. The abscess usually shows high signal intensity on T2‐weighted images and low signal intensity on T1‐weighted images.^11^, ^12^ In contrast, cystic adenomyosis, which is an important differential diagnosis of cystic lesions within the myometrium, also presents with localized fluid collection. The typical findings in cystic adenomyosis include fluid content exhibiting high signal intensity on T1‐weighted images and the surrounding myometrial wall exhibiting distinct low signal intensity on T2‐weighted images.^13^ However, lesions sometimes reveal no specific imaging features that can reliably distinguish an abscess from cystic adenomyosis. In addition to MRI, clinical characteristics are important. The two cases in this study share some clinical characteristics. First, both patients had a medical history of intrauterine manipulation, including embryo transfer procedures and transcervical resectoscopy for the removal of endometrial polyps and myomas. These manipulations might have introduced infection and/or inflammation. Second, both patients had a history of recurrent lower abdominal pain caused by intrauterine infections during embryo transfer procedures. These clinical findings are not specific but may suggest the potential causes for abscess formation.

Both patients underwent adenomyomectomy for fertility preservation. Two types of adenomyomectomy exist: partial reduction and complete excision. In both patients, complete excision was performed via laparotomy.^14^ In case 1, a subsequent pregnancy was achieved; however, it was complicated by placenta accreta spectrum and uterine rupture. Although statistical data are limited, the reported incidence of placenta accreta spectrum after uterine adenomyomectomy is 9.1%,^15^ and that of uterine rupture is 4.5%.^15^ In the patient who achieved pregnancy, additional disadvantages, such as impaired myometrial function or vulnerability of the myometrium to infection, might have been present. Therefore, in the future, before performing adenomyomectomy, careful assessment to identify whether adenomyosis lesions are associated with infection and abscess formation during the perinatal period is essential, thus accumulating more cases for further investigation.

In conclusion, we encountered two cases of adenomyosis with abscess formation. In patients with cysts associated with adenomyosis and medical history of intrauterine manipulations and infections, we should consider the presence of adenomyosis with abscess formation. Adenomyomectomy can be performed in such cases to preserve fertility.

## CONFLICT OF INTEREST STATEMENT

None.

## Data Availability

The data that support the findings of this study are available on request from the corresponding author. The data are not publicly available due to privacy or ethical restrictions. We are unable to share certain data publicly due to the sensitive nature of the information involved. The article in question contains information and images extracted from a clinical history, which are bound by strict confidentiality. Therefore, any data or information that could potentially compromise the privacy or confidentiality of a patient is strictly safeguarded. Should further information or access to specific data be needed, we kindly suggest reaching out to the corresponding author or institution directly.
